# Supplementary material for: Nursing perspectives on advancing language access in the emergency department: A qualitative study
Source: PLoS One. 2025 Nov 20;20(11):e0336525. doi: 10.1371/journal.pone.0336525 (PMC12633926; doi:10.1371/journal.pone.0336525)
Supplement: S1 File — (DOCX) [file pone.0336525.s001.docx]

Advancing Language Access in the Emergency Department through Implementation Science: A Nursing Perspective

(AFTER the study has been explained and the subject has agreed to participate.)

Thank you so much for your time today. We really appreciate your participation.

Just to review-- we are here to ask you some **open-ended questions** about interpreter services utilization and working with limited English proficiency (LEP) patients in the emergency department. This interview will last about **30-45 minutes** depending on how much you would like to share. We are interested in learning more about your impressions of language services at Olive View so that we can improve our services and provide high quality care to patients with limited English proficiency.

I will be **recording our interview** in order to not lose any valuable details you share with me, however, the recording will not be shared with anyone else beyond our research team. What you share with us will remain anonymous. Thank you again for sharing your knowledge here.

Do you have any questions before we begin?

**Demographic Data Collection**

1. Title in the emergency department:
   1. RN
   2. NA
   3. NP
2. How many years have you worked in health care? _______
3. How many years have you worked in your current position? _____
4. Do you have a bilingual certification for LA County? Y/N, If yes, what language? ______

**Qualitative Interview Guide**

1. To start, please think about the last time you had a patient that did not speak the same language as you. Could you please walk me through the process of how you usually approach a patient whose preferred language is not English?

a. What types of language services are available to you? How do you contact them? Is it easy or difficult?

b. How do you feel about using language services?

c. How do you identify patients that are LEP and require interpreter services?

d. How do you communicate with physicians, other staff, that patient requires interpreter services?

2. How does your process or decision for contacting an interpreter compare during different times within the patient visit?

a. Triage

b. Initial Assessment

c. Discharge

d. Does it differ by acuity? (i.e. if they are in critical condition vs stable)

3. How important do you think it is to contact an interpreter for cases where you don’t speak the same language as the patient?

a. What happens when you don’t contact an interpreter?

b. How do you feel about cases where you don't have an interpreter?

c. How much does your supervisor/administration expect/support working with interpreters? How about your colleagues?

d. To what extent do your co-workers utilize language services?

e. To what extend do you co-workers use their own limited non-English ability to communicate with LEP patients?

4. What problems have you encountered working with the interpreters? Contacting them? Working with them?

a. What would help you do contact an interpreter?

1. What would make it easier to work with an interpreter?
2. Are there any problems that you’ve seen specific to written translations? (i.e. discharge instructions). How have you overcome them?

5. What do you know about Olive View/DHS policy on language services?

a. Have you ever been trained on this?

b. What do you know about language access laws in general? For the state of California or nationally?

c. What do you think about these policies or laws?

6. If you could make any changes to the language services offerings at Olive View what would they be?

a. Do you have any specific ways you’ve learned or come up with that might help others to work better or more efficiently with language services?

b. Is there anything else you'd like to share with us about this topic before we close the interview?
