# Supplementary material for: Nursing perspectives on advancing language access in the emergency department: A qualitative study
Source: PLoS One. 2025 Nov 20;20(11):e0336525. doi: 10.1371/journal.pone.0336525 (PMC12633926; doi:10.1371/journal.pone.0336525)
Supplement: Attachment — Submitted filename: PLOS One Response Letter 25AUG05.docx [file pone.0336525.s003.docx]

**UNIVERSITY OF CALIFORNIA, LOS ANGELES	UCLA**

| BERKELEY • DAVIS • IRVINE • LOS ANGELES • RIVERSIDE • SAN DIEGO • SAN FRANCISCO |  | SANTA BARBARA • SANTA CRUZ |
| --- | --- | --- |

David Geffen School of Medicine at UCLA

Department of Emergency Medicine

Olive View-UCLA Medical Center

14445 Olive View Drive, North Annex

Sylmar CA 91342-1437

Tel:	(747) 210-3107

August 5, 2025

Editor

PLOS ONE

Dear Editor,

Please find attached our revised manuscript, "Nursing Perspectives on Advancing Language Access in the Emergency Department: A Qualitative Study," submitted for consideration in *PLOS ONE.* We thank the reviewers for their insightful feedback. We have addressed all requested changes and have listed our response below as requested and believe the manuscript is significantly improved. We are available to provide any further revisions or information if needed.

Please see the list of requested changes and author responses below:

1. Please note that funding information should not appear in the Acknowledgments section/Funding section or Any other areas of your Manuscript. We will only publish funding information present in the Funding Statement section of the online submission form. Please remove any funding-related text from the manuscript.

**Statement on funding in the acknowledgments section has been removed.**

2. Please provide additional details regarding participant consent. In the Methods section, please ensure that you have specified (1) whether consent was informed and (2) what type you obtained (for instance, written or verbal). If your study included minors, state whether you obtained consent from parents or guardians. If the need for consent was waived by the ethics committee, please include this information.

Details regarding consent added in the methods section.

3. Please amend your current ethics statement to include the full name of the ethics committee/institutional review board(s) that approved your specific study. 

**Full names have been added to the manuscript**.

4. We note that there were multiple versions of [ Manuscript ] in your submission's file inventory. We have removed the older file(s), retaining the most recent version(s) for editorial review. Please double check your submission file inventory and let us know if any files appear to be outdated or absent.

**The attached files appear to be correct.**


5. Please ensure that you refer to Table 2 & 3 in your text as, if accepted, production will need this reference to link the reader to the Table.

**Tables have been referred to in the text.**

Thank you very much for your consideration.

Sincerely,

Daniel Cordova, MD
